# Editorial

**Published:** 1872-02

**Authors:** 


### Editorial.

### *At Last*

We believe the season of vexatious delays is over. The April No. will see us up to time once more. The delay has been partly due to the fact that many of our most valued and able correspondents were burned out in the great fire and have not yet secured the *pou sto*—but they pledge us abundant material in the near future. Everything connected with the material relations of the JOURNAL had to be re-created. Neither publishers nor printers were willing that the CHICAGO MEDICAL JOURNAL, either in typography, paper or mechanical execution, should be inferior to itself before the fire. Improvement is to be the order of the day, in this as in all about us in our great city. Our subscribers must admit that thus far it is every way satisfactory. Now then—help us not only with your subscriptions, but your written contributions. Give us variety of interest, and we will sandwich it with such wisdom as we can command.

### *The Modern Hospital*

There is now some prospect will find a local habitation and a name in our city. Spacious grounds will be secured, rather than castellated citadels of disease and death, crowded by private residences and bounded by narrow streets. The efficient and clear-headed Superintendent of Public Charities, Dr. BEN C. MILLER, has taken up the work, and success is already nearly certain. He has our most earnest encouragement if he needs it (as he does not) and our most fervent wishes for his speedy success in giving Chicago a Hospital worthy of the city and the present age.

In this connection it is well to refer to the Hotel Dieu, of Paris, one of the most ornamental of the public buildings of that city, erected at a most enormous cost, with the intention of furnishing 800 beds, which has been unanimously condemned by the Society of Hospital Physicians and Surgeons as not in accordance with the present state of scientific and hygienic knowledge.

### *American Medical Association—Annual Meeting.*

The twenty-third annual session of the Association will be held in Philadelphia, May 7, 1872, at eleven o'clock A. M. Reports will be made by committees on subjects specially assigned, and other business will be transacted as in previous sessions. Physicians desiring to present papers before the Association should observe the following rules :

"Papers appropriate to the several sections, in order to secure consideration and action, must be sent to the secretary of the appropriate section at least one month before the meeting which is to act upon them. It shall be the duty of the secretary to whom such papers are sent to examine them with care, and, with the advice of the chairman of his section, to determine the time and order of their presentation, and give due notice of the same."

The following are the secretaries of sections :

*Chemistry and Materia Medica*—Ephraim Cutter, Boston.

*Practice of Medicine and Obstetrics*—Benj. F. Dawson, New York.

*Surgery and Anatomy*—W. F. Peck, Davenport, Ia.

*Medical Jurisprudence, Hygiene, and Physiology*—E. L. Howard, Baltimore.

*Psychology*—John Curwen, Harrisburg.

Secretaries of all medical organizations are requested to forward lists of their delegates, as soon as elected, to the permanent secretary. Other information can be obtained of the permanent secretary, Dr. W. B. Atkinson, 1400 Pine Street, Philadelphia. Railroad and hotel arrangements announced at an early day.

